# Involvement of Phosphatases in Proliferation, Maturation, and Hemoglobinization of Developing Erythroid Cells

**DOI:** 10.1155/2011/860985

**Published:** 2011-07-14

**Authors:** Eitan Fibach

**Affiliations:** Department of Hematology, Hadassah-Hebrew University Medical Center, Jerusalem 91120, Israel

## Abstract

Production of RBCs is triggered by the action of erythropoietin (Epo) through its binding to surface receptors (Epo-R) on erythroid precursors in the bone marrow. The intensity and the duration of the Epo signal are regulated by several factors, including the balance between the activities of kinesase and phosphatases. The Epo signal determines the proliferation and maturation of the precursors into hemoglobin (Hb)-containing RBCs. The activity of various protein tyrosine phosphatases, including those involved in the Epo pathway, can be inhibited by sodium orthovanadate (Na_3_VO_4_, vanadate). Adding vanadate to cultured erythroid precursors of normal donors and patients with **β**-thalassemia enhanced cell proliferation and arrested maturation. This was associated with an increased production of fetal hemoglobin (HbF). Increased HbF in patients with **β**-hemoglobinopathies (**β**-thalassemia and sickle cell disease) ameliorates the clinical symptoms of the disease. These results raise the possibility that specific and nontoxic inhibitors of phosphatases may be considered as a therapeutic modality for elevating HbF in patients with **β**-hemoglobinopathies as well as for intensifying the Epo response in other forms of anemia.

## 1. Epo and Proliferation/Maturation of Erythroid Precursors

Production of RBCs (erythropoiesis) in the bone marrow depends on the glycoprotein hormone erythropoietin (Epo), which prevents apoptosis and stimulates proliferation of erythroid precursors [1]. The activity of Epo is mediated through its binding to specific surface receptors (Epo-R) [2]. Epo binding induces receptor homodimerization and the initiation of a stepwise signal transduction process [3]. One of the earliest responses detected within cells upon ligand-induced homodimerization is a transient increase in tyrosine phosphorylation of cellular proteins including the receptor. Since the EPO-R, like other hematopoietic cytokine receptors, lacks intrinsic enzymatic activity, the receptor must associate with and activate protein tyrosine kinases in order to transmit a signal.

Upon Epo binding, EpoR undergoes phosphorylation on tyrosine residues in the cytoplasmic domain and thereby recruits different SH2-containing signaling molecules, such as the STATs, Shc, SHP-2, and the p85 regulatory subunit of PI3K to activate various signal transduction pathways, most of which are shared with other members of the cytokine receptor family [4].

Four tyrosine phosphatases are involved in regulating erythropoiesis. The SH2-domain containing phosphatases, Shp1 and Shp2, cluster designation 45 (CD45) and protein tyrosine phosphatase 1B (PTP-1B) [5]—all modulate activity of JAK2 kinase. Shp1 associates with Y429 and Y431 [6] and Shp2 to Y401 of the EPO-R [7]. Several studies have shown that Shp2 is a robust substrate for tyrosine phosphorylation by EPO-R, which leads to the recruitment of Grb2-Sos [8]. Impaired maturation of B^−^ and T^−^ cells was described originally in CD45-knockout mice and further analysis revealed that CD45 is a JAK phosphatase [9]. Study of the related tyrosine phosphatases, PTP-1B and T^−^ cell tyrosine phosphatase (TC-PTP) reveals that these enzymes trap substrates with a dityrosine motif. PTP-1B targets Tyk2 and JAK2 [10] whereas the related TC-PTP binds JAK1 and JAK3 [11]. These data indicate that these tyrosine phosphatases have unique reactivity towards JAK kinase dephosphorylation.

Another manner of desensitization involves the regulation of metabolic intermediates of inositol. Phosphatidylinositol (3,4,5)-trisphosphate activates many crucial signaling events. Several enzymes dephosphorylate it, including phosphatase and tensin homolog (which dephosphorylate the 3′ phosphate) and SH2-inositol phosphatase, SHIP-1 and SHIP-2 (which dephosphorylate the 5′ phosphate). SHIP-1 is recruited to the EPO-R [12], and SHIP-1 null mice show elevated formation of erythroid progenitors in bone marrow [13]. SHIP-1 negatively regulates B^−^ cell, macrophage, and mast cell signal transduction pathways (reviewed in [14]). Further analysis of the erythroid lineage of SHIP-1-deficient mice is needed to determine whether loss of SHIP-1 affects Erk1/2 and PKB/Akt activation, as postulated in other lineages.

The activity of Epo is mediated through its binding to specific surface receptors (Epo-R; [2]). Epo binding induces receptor homodimerization and the initiation of a stepwise signal transduction process [3]. The Epo signal is regulated by several factors. (A) The concentration of Epo. Epo is produced by the kidneys in response to hypoxia [1], secreted into the blood stream, and interacts with erythroid progenitor/precursor cells in the bone marrow. Physiologically, fluctuations in the level of Epo regulate the RBC number under conditions of hypoxia such as at high altitudes. Pathologically, increased Epo causes secondary polycythemia [15] whereas decreased production of Epo causes anemia (e.g., in patients with chronic renal failure) [16]. (B) The density of Epo-R on erythroid precursors that is downregulated during erythroid maturation [17]. (C) The stimulating activity of kinases that induce tyrosine phosphorylation of various proteins in the Epo signaling pathway [18]. A single-base somatic mutation in the gene of one of these kinases, Janus kinase 2 (JAK2), was reported to be responsible for autostimulation of the pathway, causing Epo-independent growth of erythroid cells in polycythemia vera [19]. (D) The inhibitory activity of phosphatases that downregulates the signal, causing it to be transient and dependent on continuous Epo binding [20].

The intensity and duration of the ERO signal regulate the balance between the rates of proliferation and maturation of the erythroid precursors. At early stages of development, when the signal is intense due to abundance of Epo-R [17], proliferation prevails. As the density of the receptors drops, the intensity of the signal weakens, and maturation overcomes. The concentration of Epo, as well as other cytokines such as the stem cell factor (SCF), also affects the intensity of the signal and, thereby, the rate of maturation. High concentrations of these cytokines delay maturation, increase proliferation/amplification, and result in a high cell yield [21]. The intensity of the Epo signal also depends on the balance between the activities of kinases and phosphatases in the Epo pathway [18].

## 2. Inhibition of Phosphatases: Effect on Proliferation/Maturation of Erythroid Precursors

The activity of various protein tyrosine phosphatases, including those involved in the Epo pathway, can be inhibited by sodium orthovanadate (Na_3_VO_4_, vanadate; [22]) and thereby downregulates the signal. Pervanadate compounds are the most potent inhibitors of cellular protein tyrosine phosphatases [23]. As oxidants, their mode of action probably involves oxidation of a cysteine residue in the active site of tyrosine phosphatases [24]. Treatment of cells with vanadate perturbs the equilibrium of tyrosine phosphorylation/dephosphorylation, causing elevated protein phosphorylation. In the insulin receptor, vanadate mimics the lipogenic effect of insulin via activation of the insulin receptor tyrosine kinase [23], resulting in the phosphorylation of cellular substrates [25]. In T^−^ cells, it was shown to mimic events mediated by activation of the T^−^ cell antigen receptor [26, 27] and to activate intracellular signaling pathways mediated by the interleukin-2 receptor [28], STAT1 [29] and the MAP kinase pathway [30].

It was reported that treatment with vanadate of the Epo-dependent HCD57 murine cell line resulted in increased tyrosine protein phosphorylation. Vanadate acted synergistically with Epo to stimulate DNA synthesis and prevented apoptosis following Epo withdrawal without promoting proliferation [31, 32]. It also delayed apoptosis in primary human erythroid progenitors [31]. Vanadate was also shown to act on normal erythroid progenitors as a phosphatase inhibitor that potentiates the kinase activity induced by Epo and SCF. This function was, however, reduced in polycythemia vera cells [33].

We investigated the effect of vanadate on the proliferation and maturation of human erythroid precursors in culture [34]. For this purpose we used the two-phase liquid culture protocol [35]. In the first, Epo-independent, phase of this protocol, peripheral blood mononuclear cells are cultured for 1 week with various growth factors but in the absence of Epo. During this phase, early erythroid committed progenitors, erythroid burst-forming units, proliferate and differentiate into late, erythroid colony forming unit-like, Epo-dependent, progenitors. In the second phase, the latter cells, cultured in an Epo-supplemented medium, continue to proliferate and differentiate, eventually maturing into hemoglobin (Hb)-containing orthochromatic normoblasts and enucleated erythrocytes. When vanadate was added to cells derived from normal donors, cell proliferation was enhanced as indicated by studying the growth kinetics and the distribution in the cell cycle phases. On the contrary, maturation was arrested, as indicated by cell morphology, the rate of appearance of Hb-containing cells, and the pattern of expression of surface antigens (CD117, CD71, and glycophorin A) [34].

## 3. Maturation Arrest in Erythroid Precursors

Maturation arrest of hematopoietic precursors occurs in acute leukemia. Maturation arrest of erythroid precursors is a common phenomenon in *β*-thalassemia. In this disease, due to hereditary mutations, the expression of the *β*-globin gene is reduced or abolished. This results in a relative high content of *α*-globin, which forms tetramers that precipitate and damage the erythroid cell. Although maturation arrest causes expansion of the erythroid precursors in the bone marrow and in extramedullary sites, such as the liver and spleen, the output of mature RBCs is decreased due to ineffective erythropoiesis. The latter involves premature apoptosis of erythroid precursors. Although this condition was extensively studied in patients [36] and in mouse models of *β*-thalassemia [37, 38], its causes in thalassemia are not entirely clear. It could be related to the following: (A) the reduced production of *β*-globin which, consequently, results in imbalanced production of *α*-globin, (B) reduced heme synthesis which modifies the activity of the heme-regulated eIF2 alpha kinase which controls protein synthesis by phosphorylating the *α*-subunit of eukaryotic translational initiation factor 2 (eIF2 alpha) [39], (C) inefficient elimination of free radicals, which are increased in thalassemia as a result of iron overload [40], (D) the hypoxia-induced overproduction of Epo which is the result of the severe, chronic anemia. Serum-Epo is high in these patients although it does not reach the high levels corresponding to the degree of anemia [41] (as compared to other forms of anemia such as aplastic anemia). This could be due to increased consumption of Epo by the large erythroid mass. The increased Epo signal may lead to increased proliferation of erythroid precursors bearing a phosphorylated form of JAK2, which, in pathological conditions, may lead to a delay in cell maturation.

## 4. Inhibition of Phosphatases: Effect on Hemoglobin Production

We have found that addition of vanadate to cultures of erythroid precursors derived from normal donors as well as from patients with *β*-thalassemia increased the proportion of fetal hemoglobin (HbF) compared to untreated cells [34]. HbF, which is composed of two chains of alpha-globin and two chains of gamma-globin (*α*_*2*_ *γ*_*2*_) is the major Hb during embryonic life. It is replaced after birth by adult Hb (HbA; *α*_*2*_ *β*_*2*_, [42]). This Hb switch is recapitulated to some extent postnatally during the development of erythroid cell in the bone marrow; HbF production is relatively abundant in early precursors, and as the cells mature, progressively decreases due to rapid synthesis of HbA [43], suggesting that a strong Epo signal favors HbF production. Elevated levels of HbF in post-natal life may be acquired, such as in juvenile myelomonocytic leukemia [44] or during acute erythropoietic stress [45], and are frequently observed in inherited blood disorders such as *β*-thalassemia and sickle cell anemia [46]. Increased HbF in these diseases ameliorates the clinical symptoms of the underlying disease [47, 48]. In *β*-thalassemia, elevated HbF compensates partially for the deficiency in *β*-globin chains and balances the excess of *α*-globin chains. In sickle cell anemia, not only do HbF-containing cells have a lower concentration of sickle Hb, but HbF inhibits polymerization of this Hb directly, accounting for the lower propensity of such cells to undergo sickling [49–51]. Various agents have been shown to augment HbF production, and one of them, hydroxyurea, is currently in clinical use for treatment of these diseases [52].

The mechanisms by which drugs stimulate HbF are not known. Two broad hypotheses have been explored. One is based on drug-induced modifications of the DNA due to hypomethylation of globin promoter regions [53], inhibition of histone deacetylases [54], or activation of responsive regions, such as following binding of butyrates to a specific region of the ^A^ *γ*-globin promoter [55]. The other mechanism involves modification of the cell cycle kinetics [56] and the rate of differentiation of erythroid progenitors [57, 58]. Observations of elevated HbF during erythropoietic stress [45] suggest that the rate of erythroid maturation and the intensity and duration of the Epo signal affect HbF production. Some studies [59, 60], but not all [61–63], showed that high-dose Epo treatment of primates [64, 65] and patients with *β*-hemoglobinopathies caused an elevation in HbF. We have previously shown that culture of erythroid cells in the continuous presence of low Epo reduced cell yield but did not affect the proportion of HbF. However, reducing Epo levels midway through the culture period, lowered cell yield, accelerated maturation, shortened the period of HbA production, and, consequently, increased the proportion of HbF [21]. In another study, we found that supplying early erythroid cultures with exogenous hemin (heme chloride) resulted in high HbF in the mature cells [66]. The effect of hemin, which is a rate-limiting factor for hemoglobinization in early precursors, may be related to the finding that when supplied with exogenous hemin, the precursors initiated Hb production earlier with HbF predominating. SCF, which delays cell maturation, was also shown to enhance HbF production in cultures of erythroid cells [8, 67–69]. A direct involvement of phosphatases in stimulation of HbF accumulation was reported by Aerbajinai et al. who examined the pathway of *γ*-globin synthesis stimulation by SCF. It was found that COUP-TFII a repressor of *γ*-globin gene was suppressed by SCF through phosphorylation of serine/threonine phosphatase (PP2A) and correlated well with HbF induction [70].

**Figure 1:**
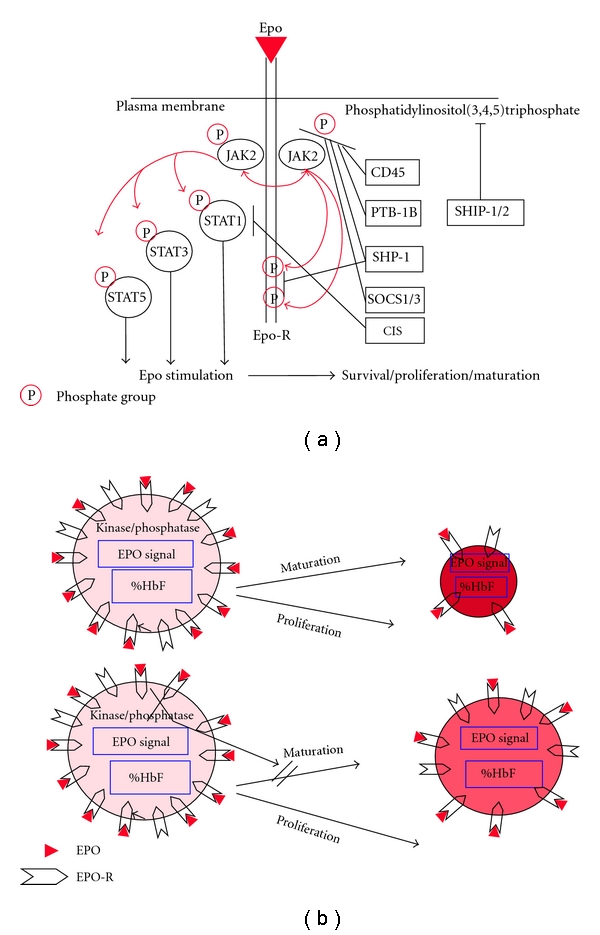
The effect of phosphatases on the proliferation/maturation of erythroid precursors and their fetal hemoglobin content. The kinetics of maturation and hemoglobinization in developing erythroid cells depends on the Epo signal. This is modulated by the level of erythropoietin (Epo), produced in response to hypoxic conditions, the number of erythropoietin-receptors (Epo-R), and the balance in the activities of kinases and phosphatases. (a) Binding of Epo causes Epo-R homodimerization and activation of the receptor-bound tyrosine kinase JAK2. Transphosphorylation of JAK2 results in activation of JAK2 and tyrosine phosphorylation (symbolized by the red arrows) of proteins (e.g., STATs), including the cytoplasmic domain of the EPO-R. Negative regulation of the Epo stimulus involves the tyrosine phosphatases CD45, PTP-1B, and Shp1. CIS and SOCS3 compete for STAT5 binding at Y401 whereas SOCS1 and SOCS3 bind to the activation loop of JAK2. Regulation of phosphoinositide metabolism by PI 3-kinase and SHIP1 is also indicated. Activation of the EPO-R supports survival, proliferation, and maturation of erythroid precursors. (b) Early erythroid precursors (left) carrying a large number of Epo-R are stimulated by Epo. The strong Epo-signal drives the cells to undergo proliferation and maturation. As maturation proceeds (right), the cell size and the number of Epo-R decrease, and while total Hb accumulates (red color), the proportion of HbF (%HbF) decreases. Addition of a phosphatase inhibitor (e.g., vanadate) to early erythroid precursors blocks phosphatase activity (lower panel, marked by X), resulting in continuous high Epo signaling and inhibition of cell maturation. The latter is accompanied by a relatively high proportion of HbF.

In summary, the phosphatase-inhibitory compound vanadate delays maturation of erythroid precursors and potentiates their ability to produce HbF (Figure 1). The latter effect may be beneficial for patients with *β*-hemoglobinopathies, since increased level of this Hb was found to ameliorate the clinical symptoms of the underlying disease. Vanadate, in the form of sodium metavanadate, has been tested in clinical trials for treating both insulin- and noninsulin-dependent diabetes mellitus [71, 72]. In *in vitro* studies vanadate was shown to reduce the number and Hb content of erythroid cells [34], and therefore it is not suitable for treatment of anemic patients. However, other more specific and less toxic inhibitors of phosphatases may be considered as a new therapeutic modality for elevating HbF in patients with *β*-hemoglobinopathies as well as intensifying the Epo response in other forms of anemia.

## References

[1] Jelkmann W (1992). Erythropoietin: structure, control of production, and function. *Physiological Reviews*.

[2] Broudy VC, Lin N, Brice M, Nakamoto B, Papayannopoulou T (1991). Erythropoietin receptor characteristics on primary human erythroid cells. *Blood*.

[3] Watowich SS, Liu KD, Xie X (1999). Oligomerization and scaffolding functions of the erythropoietin receptor cytoplasmic tail. *Journal of Biological Chemistry*.

[4] Constantinescu SN, Ghaffari S, Lodish HF (1999). The erythropoietin receptor: structure, activation and intracellular signal transduction. *Trends in Endocrinology and Metabolism*.

[5] Frangioni JV, Beahm PH, Shifrin V, Jost CA, Neel BG (1992). The nontransmembrane tyrosine phosphatase PTP-1B localizes to the endoplasmic reticulum via its 35 amino acid C-terminal sequence. *Cell*.

[6] Klingmuller U, Lorenz U, Cantley LC, Neel BG, Lodish HF (1995). Specific recruitment of SH-PTP1 to the erythropoietin receptor causes inactivation of JAK2 and termination of proliferative signals. *Cell*.

[7] Tauchi T, Damen JE, Toyama K, Feng GS, Broxmeyer HE, Krystal G (1996). Tyrosine 425 within the activated erythropoietin receptor binds Syp, reduces the erythropoietin required for Syp tyrosine phosphorylation, and promotes mitogenesis. *Blood*.

[8] Peschle C, Gabbianelli M, Testa U (1993). c-kit ligand reactivates fetal hemoglobin synthesis in serum-free culture of stringently purified normal adult burst-forming unit-erythroid. *Blood*.

[9] Irie-Sasaki J, Sasaki T, Matsumoto W (2001). CD45 is a JAK phosphatase and negatively regulates cytokine receptor signalling. *Nature*.

[10] Myers MP, Andersen JN, Cheng A (2001). TYK2 and JAK2 are substrates of protein-tyrosine phosphatase 1B. *Journal of Biological Chemistry*.

[11] Simoncic PD, Lee-Loy A, Barber DL, Tremblay ML, McGlade CJ (2002). The T cell protein tyrosine phosphatase is a negative regulator of janus family kinases 1 and 3. *Current Biology*.

[12] Mason JM, Beattie BK, Liu Q, Dumont DJ, Barber DL (2000). The SH2 inositol 5-phosphatase Ship1 is recruited in an SH2-dependent manner to the erythropoietin receptor. *Journal of Biological Chemistry*.

[13] Helgason CD, Damen JE, Rosten P (1998). Targeted disruption of SHIP leads to hemopoietic perturbations, lung pathology, and a shortened life span. *Genes and Development*.

[14] Kalesnikoff J, Sly LM, Hughes MR (2003). The role of SHIP in cytokine-induced signaling. *Reviews of Physiology, Biochemistry and Pharmacology*.

[15] Lee FS (2008). Genetic causes of erythrocytosis and the oxygen-sensing pathway. *Blood Reviews*.

[16] Lankhorst CE, Wish JB (2010). Anemia in renal disease: diagnosis and management. *Blood Reviews*.

[17] Shinjo K, Takeshita A, Higuchi M, Ohnishi K, Ohno R (1997). Erythropoietin receptor expression on human bone marrow erythroid precursor cells by a newly-devised quantitative flow-cytometric assay. *British Journal of Haematology*.

[18] Klingmuller U (1997). The role of tyrosine phosphorylation in proliferation and maturation of erythroid progenitor cells—signals emanating from the erythropoietin receptor. *European Journal of Biochemistry*.

[19] Levine RL, Wadleigh M, Cools J (2005). Activating mutation in the tyrosine kinase JAK2 in polycythemia vera, essential thrombocythemia, and myeloid metaplasia with myelofibrosis. *Cancer Cell*.

[20] Xu D, Qu CK (2008). Protein tyrosine phosphatases in the JAK/STAT pathway. *Frontiers in Bioscience*.

[21] Fibach E, Schechter AN, Noguchi CT, Rodgers GP (1994). Reducing erythropoietin in cultures of human erythroid precursors elevates the proportion of fetal haemoglobin. *British Journal of Haematology*.

[22] Cohen J, Altaratz H, Zick Y, Klingmuller U, Neumann D (1997). Phosphorylation of erythropoietin receptors in the endoplasmic reticulum by pervanadate-mediated inhibition of tyrosine phosphatases. *Biochemical Journal*.

[23] Fantus IG, Kadota S, Deragon G, Foster B, Posner BI (1989). Pervanadate [peroxide(s) of vanadate] mimics insulin action in rat adipocytes via activation of the insulin receptor tyrosine kinase. *Biochemistry*.

[24] Guan KL, Dixon JE (1990). Protein tyrosine phosphatase activity of an essential virulence determinant in Yersinia. *Science*.

[25] Heffetz D, Bushkin I, Dror R, Zick Y (1990). The insulinomimetic agents H2O2 and vanadate stimulate protein tyrosine phosphorylation in intact cells. *Journal of Biological Chemistry*.

[26] O’Shea JJ, McVicar DW, Bailey TL, Burns C, Smyth MJ (1992). Activation of human peripheral blood T lymphocytes by pharmacological induction of protein-tyrosine phosphorylation. *Proceedings of the National Academy of Sciences of the United States of America*.

[27] Secrist JP, Burns LA, Karnitz L, Koretzky GA, Abraham RT (1993). Stimulatory effects of the protein tyrosine phosphatase inhibitor, pervanadate, on T-cell activation events. *Journal of Biological Chemistry*.

[28] Evans GA, Garcia GG, Erwin R, Howard OMZ, Farrar WL (1994). Pervanadate simulates the effects of interleukin-2 (IL-2) in human T cells and provides evidence for the activation of two distinct tyrosine kinase pathways by IL-2. *Journal of Biological Chemistry*.

[29] Tourkine N, Schindler C, Larose M, Houdebine LM (1995). Activation of STAT factors by prolactin, interferon-gamma, growth hormones, and a tyrosine phosphatase inhibitor in rabbit primary mammary epithelial cells. *Journal of Biological Chemistry*.

[30] Zhao Z, Tan Z, Diltz CD, You M, Fischer EH (1996). Activation of mitogen-activated protein (MAP) kinase pathway by pervanadate, a potent inhibitor of tyrosine phosphatases. *Journal of Biological Chemistry*.

[31] Spivak JL, Fisher J, Isaacs MA, Hankins WD (1992). Protein kinases and phosphatases are involved in erythropoietin-mediated signal transduction. *Experimental Hematology*.

[32] Lawson AE, Bao H, Wickrema A, Jacobs-Helber SM, Sawyer ST (2000). Phosphatase inhibition promotes antiapoptotic but not proliferative signaling pathways in erythropoietin-dependent HCD57 cells. *Blood*.

[33] Dai CH, Krantz SB, Sawyer ST (1997). Polycythemia vera. V. Enhanced proliferation and phosphorylation due to vanadate are diminished in polycythemia vera erythroid progenitor cells: a possible defect of phosphatase activity in polycythemia vera. *Blood*.

[34] Amoyal I, Prus E, Fibach E (2007). Vanadate elevates fetal hemoglobin in human erythroid precursors by inhibiting cell maturation. *Experimental Biology and Medicine*.

[35] Fibach E, Manor D, Oppenheim A, Rachmilewitz EA (1989). Proliferation and maturation of human erythroid progenitors in liquid culture. *Blood*.

[36] Centis F, Tabellini L, Lucarelli G (2000). The importance of erythroid expansion in determining the extent of apoptosis in erythroid precursors in patients with beta-thalassemia major. *Blood*.

[37] Libani IV, Guy EC, Melchiori L (2008). Decreased differentiation of erythroid cells exacerbates ineffective erythropoiesis in beta-thalassemia. *Blood*.

[38] Rivella S (2009). Ineffective erythropoiesis and thalassemias. *Current Opinion in Hematology*.

[39] Han AP, Fleming MD, Chen JJ (2005). Heme-regulated eIF2alpha kinase modifies the phenotypic severity of murine models of erythropoietic protoporphyria and beta-thalassemia. *Journal of Clinical Investigation*.

[40] Fibach E, Rachmilewitz E (2008). The role of oxidative stress in hemolytic anemia. *Current Molecular Medicine*.

[41] Manor D, Fibach E, Goldfarb A, Rachmilewitz EA (1986). Erythropoietin activity in the serum of beta thalassemic patients. *Scandinavian Journal of Haematology*.

[42] Stamatoyannopoulos G, Grosveld F (2001). *Hemoglobin Switching*.

[43] Dalyot N, Fibach E, Rachmilewitz EA, Oppenheim A (1992). Adult and neonatal patterns of human globin gene expression are recapitulated in liquid cultures. *Experimental Hematology*.

[44] Weatherall DJ, Edwards JA, Donohoe WT (1968). Haemoglobin and red cell enzyme changes in juvenile myeloid leukaemia. *British Medical Journal*.

[45] Alter BP (1979). Fetal erythropoiesis in stress hematopoiesis. *Experimental Hematology*.

[46] Sankaran VG, Nathan DG (2010). Reversing the hemoglobin switch. *The New England Journal of Medicine*.

[47] Al-Awamy BH, Niazi GA, el-Mouzan MI (1986). Relationship of haemoglobin F and alpha thalassaemia to severity of sickle-cell anaemia in the Eastern Province of Saudi Arabia. *Annals of Tropical Paediatrics*.

[48] Haghshenass M, Ismail-Beigi F, Clegg JB, Weatherall DJ (1977). Mild sickle cell anaemia in Iran associated with high levels of fetal haemoglobin. *Journal of Medical Genetics*.

[49] Benesch RE, Edalji R, Benesch R, Kwong S (1980). Solubilization of hemoglobin S by other hemoglobins. *Proceedings of the National Academy of Sciences of the United States of America*.

[50] Eaton WA, Hofrichter J (1995). The biophysics of sickle cell hydroxyurea therapy. *Science*.

[51] Noguchi CT, Rodgers GP, Serjeant G, Schechter AN (1988). Levels of fetal hemoglobin necessary for treatment of sickle cell disease. *The New England Journal of Medicine*.

[52] Rodgers GP, Rachmilewitz EA (1995). Novel treatment options in the severe beta-globin disorders. *British Journal of Haematology*.

[53] Charache S, Dover G, Smith K, Talbot CC, Moyer M, Boyer S (1983). Treatment of sickle cell anemia with 5-azacytidine results in increased fetal hemoglobin production and is associated with nonrandom hypomethylation of DNA around the gamma-delta-beta-globin gene complex. *Proceedings of the National Academy of Sciences of the United States of America*.

[54] McCaffrey PG, Newsome DA, Fibach E, Yoshida M, Su MSS (1997). Induction of gamma-globin by histone deacetylase inhibitors. *Blood*.

[55] Hudgins WR, Fibach E, Safaya S, Rieder RF, Miller AC, Samid D (1996). Transcriptional upregulation of gamma-globin by phenylbutyrate and analogous aromatic fatty acids. *Biochemical Pharmacology*.

[56] Letvin NL, Linch DC, Beardsley GP, McIntyre KW, Miller BA, Nathan DG (2005). Influence of cell cycle phase-specific agents on simian fetal hemoglobin synthesis. *The Journal of Clinical Investigation*.

[57] Humphries RK, Dover G, Young NS (1985). 5-Azacytidine acts directly on both erythroid precursors and progenitors to increase production of fetal hemoglobin. *The Journal of Clinical Investigation*.

[58] Torrealba-de Ron AT, Papayannopoulou T, Knapp MS, Fu MF, Knitter G, Stamatoyannopoulos G (1984). Perturbations in the erythroid marrow progenitor cell pools may play a role in the augmentation of HbF by 5-azacytidine. *Blood*.

[59] Breymann C, Fibach E, Visca E, Huettner C, Huch A, Huch R (1999). Induction of fetal hemoglobin synthesis with recombinant human erythropoietin in anemic patients with heterozygous beta-thalassemia during pregnancy. *Journal of Maternal-Fetal and Neonatal Medicine*.

[60] Makis A, Chaliasos N, Hatzimichael E, Bourantas KL (2001). Recombinant human erythropoietin therapy in a transfusion-dependent beta-thalassemia major patient. *Annals of Hematology*.

[61] Goldberg MA, Brugnara C, Dover GJ, Schapira L, Lacroix L, Bunn HF (1992). Hydroxyurea and erythropoietin therapy in sickle cell anemia. *Seminars in Oncology*.

[62] Olivieri NF, Freedman MH, Perrine SP (1992). Trial of recombinant human erythropoietin: three patients with thalassemia intermedia. *Blood*.

[63] Rachmilewitz EA, Goldfarb A, Dover G (1991). Administration of erythropoietin to patients with beta-thalassemia intermedia: a preliminary trial. *Blood*.

[64] Al-Khatti A, Veith RW, Papayannopoulou T, Fritsch EF, Goldwasser E, Stamatoyannopoulos G (1987). Stimulation of fetal hemoglobin synthesis by erythropoietin in baboons. *The New England Journal of Medicine*.

[65] Stamatoyannopoulos G, Umemura T, al-Khatti A (1989). Modulation of HBF production by erythropoietin. *Progress in Clinical and Biological Research*.

[66] Fibach E, Kollia P, Schechter AN, Noguchi CT, Rodgers GP (1995). Hemin-induced acceleration of hemoglobin production in immature cultured erythroid cells: preferential enhancement of fetal hemoglobin. *Blood*.

[67] Bhanu NV, Trice TA, Lee YT (2005). A sustained and pancellular reversal of gamma-globin gene silencing in adult human erythroid precursor cells. *Blood*.

[68] Miller BA, Perrine SP, Bernstein A (1992). Influence of steel factor on hemoglobin synthesis in sickle cell disease. *Blood*.

[69] Muta K, Krantz SB, Bondurant MC, Dai CH (1995). Stem cell factor retards differentiation of normal human erythroid progenitor cells while stimulating proliferation. *Blood*.

[70] Aerbajinai W, Zhu J, Kumkhaek C, Chin K, Rodgers GP (2009). SCF induces gamma-globin gene expression by regulating downstream transcription factor COUP-TFII. *Blood*.

[71] Goldfine AB, Simonson DC, Folli F, Patti ME, Kahn CR (1995). In vivo and in vitro studies of vanadate in human and rodent diabetes mellitus. *Molecular and Cellular Biochemistry*.

[72] Goldfine AB, Simonson DC, Folli F, Patti ME, Kahn CR (1995). Metabolic effects of sodium metavanadate in humans with insulin-dependent and noninsulin-dependent diabetes mellitus in vivo and in vitro studies. *Journal of Clinical Endocrinology and Metabolism*.

